# Electroacupuncture-Related Metabolic Brain Connectivity in Neuropathic Pain due to Brachial Plexus Avulsion Injury in Rats

**DOI:** 10.3389/fncir.2020.00035

**Published:** 2020-06-17

**Authors:** Ao-Lin Hou, Mou-Xiong Zheng, Xu-Yun Hua, Bei-Bei Huo, Jun Shen, Jian-Guang Xu

**Affiliations:** ^1^Shanghai Eighth People Hospital, Shanghai, China; ^2^Department of Traumatology and Orthopedics, Yueyang Hospital, Shanghai University of Traditional Chinese Medicine, Shanghai, China; ^3^School of Rehabilitation Science, Shanghai University of Traditional Chinese Medicine, Shanghai, China; ^4^Department of Orthopedics, Guanghua Hospital of Integrative Chinese and Western Medicine, Shanghai, China

**Keywords:** neuropathic pain, brachial plexus avulsion injury, electroacupuncture, thermal withdrawal latency, metabolic connectivity

## Abstract

**Objective**: The present study aimed to investigate the analgesic effect of electroacupuncture (EA) in neuropathic pain due to brachial plexus avulsion injury (BPAI) and related changes in the metabolic brain connectivity.

**Methods**: Neuropathic pain model due to BPAI was established in adult female Sprague–Dawley rats. EA stimulations (2/15 Hz, 30 min/day, 5-day intervention followed by 2-day rest in each session) were applied to the fifth–seventh cervical “Jiaji” acupoints on the noninjured side from 1st to 12th weeks following BPAI (EA group, *n* = 8). Three control groups included sham EA (nonelectrical acupuncture applied to 3 mm lateral to the real “Jiaji” acupoints), BPAI-only, and normal rats (no particular intervention; eight rats in each group). Thermal withdrawal latency (TWL) of the noninjured forepaw was regularly tested to evaluate the threshold of thermalgesia. Small animal [fluorine-18]-fluoro-2-deoxy-_D_-glucose (^18^F-FDG) PET/CT scans of brain were conducted at the end of 4th, 12th, and 16th weeks to explore metabolic alterations of brain.

**Results**: In the EA group, the TWL of the noninjured forepaw significantly decreased following BPAI and then increased following EA stimulation, compared with sham EA (*P* < 0.001). The metabolic brain connectivity among somatosensory cortex (SC), motor cortex (MC), caudate putamen (Cpu), and dorsolateral thalamus (DLT) in bilateral hemispheres decreased throughout the 16 weeks’ observation in the BPAI-only group, compared with the normal rats (*P* < 0.05). In the EA group, the strength of connectivity among the above regions were found to be increased at the end of 4th week following BPAI modeling, decreased at 12th week, and then increased again at 16th week (*P* < 0.05). The changes in metabolic connectivity were uncharacteristic and dispersed in the sham EA group.

**Conclusion**: The study revealed long-term and extensive changes of metabolic brain connectivity in EA-treated BPAI-induced neuropathic pain rats. Bilateral sensorimotor and pain-related brain regions were mainly involved in this process. It indicated that modulation of brain metabolic connectivity might be an important mechanism of analgesic effect in EA stimulation for the treatment of neuropathic pain.

## Introduction

Brachial plexus avulsion injury (BPAI), which is often caused by motorcycle accident, is one of the most devastating peripheral nerve injuries. Partial or global BPAI would lead to sensory and motor dysfunction. Neuropathic pain is another intractable problem caused by BPAI. It is reported that 70–90% BPAI patients suffer from persistent neuropathic pain (Teixeira et al., 2015). This kind of pain is usually described as hot-burning, tingling, pricking, pins-and-needles, sharp, shooting, squeezing, cold, electric, or shock-like quality of pain (Widerström-Noga, 2017). Functional and structural changes in the nervous system involve the peripheral nerve fibers, spine, brain, and several pain pathways (Teixeira et al., 2015). These factors would contribute to both development and maintenance of neuropathic pain (Gierthmühlen and Baron, 2016; Zilliox, 2017). Neuropathic pain following BPAI was reported to be largely refractory to the conventional treatment, and evidenced-based treatments for it are scarce (Parry, 1980; Teixeira et al., 2015). Investigation on the mechanisms underlying neuropathic pain due to BPAI would be necessary for seeking the solve for neuropathic pain.

Several other guidelines have focused on the clinical practice of pharmacological medicine (Attal et al., 2010). Nonpharmacological therapy, such as direct current stimulation with (or without) visual illusion, transcutaneous electrical stimulation, dorsal root entry zone lesioning, and acupuncture are recommended for neuropathic pain after spinal cord injury (Davis and Lentini, 1975; Spaić et al., 2002; Soler et al., 2010; Cohen and Mao, 2014; Ngernyam et al., 2015). However, chronic neuropathic pain is still a challenging problem (Zilliox, 2017). Acupuncture is a popular practice of traditional Chinese medicine. It modulates the flow of Qi and blood through the meridians of the body and restores the balance among five main organs (heart, liver, spleen, kidney, and lung) to maintain homeostasis (Rong et al., 2011). Acupuncture or electroacupuncture (EA) has been used as alternative therapy in the treatment of several diseases (Li et al., 2014; Lu et al., 2014). Chronic pain is one of its major indications, including inflammatory pain, cancer-related pain, visceral pain, and neuropathic pain (Ju et al., 2017; Zhang et al., 2014). However, the underlying mechanism is largely unknown.

As the development of neuroimaging technology, [fluorine-18]-fluoro-2-deoxy-_D_-glucose (^18^F-FDG) PET has been used as a method for measuring the cerebral metabolic rate of glucose from 1980 (Phelps et al., 1980). It directly measures the glucose metabolism as a marker of neural activity and reflects the whole brain activity, which is in the steady state. Compared with blood-oxygen-level-dependent imaging (BOLD) functional magnetic resonance imaging (fMRI), ^18^F-FDG-PET is less dependent on neurovascular coupling (Yakushev et al., 2017). Metabolic connectivity reflects metabolism relationships between different brain regions, which could provide a novel insight of brain connectivity (Yakushev et al., 2017). It has been used in many diseases such as Alzheimer’s disease (AD; Morbelli et al., 2012; Herholz et al., 2018), dementia (Caminiti et al., 2016), and amyotrophic lateral sclerosis (Pagani et al., 2016).

In the present study, the BPAI-induced neuropathic pain was established in rats. Sham EA, BPAI-only (model), and normal groups were set up for control. The changes in thermalgesia threshold were evaluated. Meanwhile, ^18^F-FDG traced positron emission tomography/computed tomography (PET/CT) was acquired to assess the change in brain metabolism. Metabolic connectivity was further calculated to explore the underlying mechanisms of neural activity in BPAI rats.

## Materials and Methods

### Animals

A total of 32 female Sprague–Dawley rats weighing 180–200 g were included. All the rats were provided by Shanghai Slack Laboratory Animal Limited Liability Company (Shanghai, China). They were raised under the conditions with 12-h light/dark cycle and unrestricted food or water supply. The rats were kept in cages for at least 7 days before any further intervention or assessment started. The rats were randomly assigned to four groups: the normal, model, sham EA, and EA groups (eight rats in each group).

**Figure 1:**
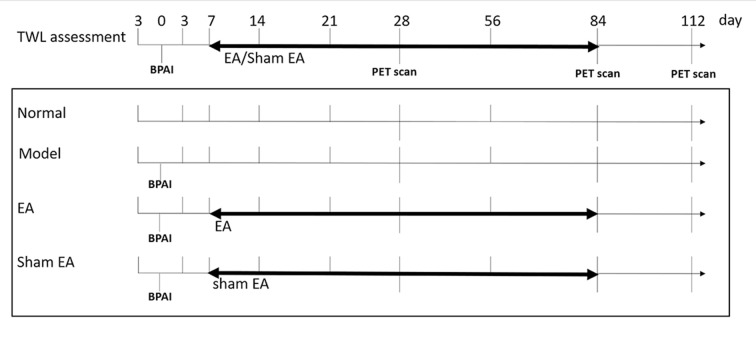
Flow chart of whole experiment. Right side brachial plexus avulsion injury (BPAI) was made on day 0. TWL assessment was conducted on 3rd day before BPAI and 3th, 7th, 14th, 21th, 28th, 56th, 84th, and 112th day following BPAI, respectively. (^18^F-FDG) PET/CT scan was performed on 28th, 84th, and 112th day following BPAI, respectively. EA/sham EA intervention was performed from 7th to 84th day, which lasted for 11 weeks. BPAI, brachial plexus avulsion; TWL, thermal withdrawal latency; EA, electroacupuncture.

All procedures were in agreement with the Guide for the Care and Use of Laboratory Animals described by the US National Institutes of Health and were approved by the Animal Ethical Committee of Shanghai University of Traditional Chinese Medicine. The flow diagram is shown in Figure 1.

### Surgical Procedure

The global BPAI (C5-T1 cervical nerve roots) procedure was performed on the right side in the rats of model, sham EA, and EA groups according to the method described in our previous study (Shen et al., 2019a). During the procedure, the rat was anesthetized *via* intraperitoneal injection with sodium pentobarbital (40 mg/kg) and then placed in a prone position on a clean operation table. An incision of 4 cm was made through the dorsal midline from the occiput to the scapular angulus superior. Under an operation microscope (magnification, 10×), longissimus capitis muscle, semispinal muscle of the neck, biventer cervicis, and complex muscle were divided. The muscles on vertebral plate and the spinous process were removed, and hemilaminectomies from C4 to T1 were performed to expose the nerve roots on the right side. The roots of C5, C6, C7, C8, and T1 nerves were identified under direct vision. Both dorsal and ventral rootlets were grasped with forceps and completely extracted from the spinal cord by traction. The rootlets and dorsal ganglions were visually confirmed to ensure a preganglionic injury. Glutin sponge was applied for hemostasis, and the incision was covered with penicillin powder to prevent infection.

### EA/Sham EA Interventions

The EA and sham EA interventions were conducted at the same time of the day since first week following BPAI. The rats were placed in a self-made immobilization apparatus, with the right hindlimb and low back exposed and the rest of the body fixed. Room temperature was controlled to 25.0 ± 1.0°C, with noise and light controlled. The rats acclimated for at least 3 days before the interventions started.

In the EA stimulation, three disposable sterile stainless-steel needles (0.3 mm in diameter and 13 mm in length; Huatuo, Suzhou Medical Appliance Factory, Suzhou, China) were inserted into the left C5–C7 “Jiaji” acupoints (EX-B2, 5 mm lateral to the left C5–C7 spinous, 3–5 mm in depth). Then, the needles’ tails were connected to the output terminal of an electrical stimulator (Huatuo SDZ-II nerve and muscle stimulator, Suzhou Medical Appliance Factory, Suzhou, China). The frequency of disperse-dense wave of EA was 2/15 Hz, and the intensity was modulated to induce slight contraction of target muscles. The intervention was performed for 30 min per day, five times per week for 11 weeks.

In the sham EA group, three needles were inserted into sham acupoints (8 mm lateral to the left C5–C7 spinous, 3–5 mm in depth) without electrical output.

### Behavioral Assessments

Thermal withdrawal latency (TWL) of the noninjured (left) forepaw was tested 3 days pre-BPAI, 3rd day, and at the end of the 1st, 2nd, 3rd, 4th, 8th, 12th, and 16th weeks after BPAI (Rodrigues-Filho et al., 2003; Challa, 2015). Before the first evaluation, the rats were trained 5 days per week for 2 weeks.

The TWL tests were performed with the Plantar Test Apparatus (Hargreaves Method) for Mice and Rats (IITC Life Science Inc., Woodland Hills, CA, United States). The rats were placed on a glass platform for 15 min before the test to ensure acclimation. Then, a high-intensity moveable radiant heat source was placed underneath. The lateral palmar surface of the noninjured (left) forepaw was exposed to the radiant heat source. The time when the rat withdraws or moves the forepaw after placement of the heat source was recorded, with shorter latencies indicating lower thermalgesia thresholds. The heat source automatically cut off at 20 s to avoid tissue damage. Five consecutive tests were conducted in each rat, with intervals of 5 min in between. The average value was recorded as the final result.

### ^18^F-FDG PET/CT Scanning

^18^F-FDG PET scanning were acquired at the end of the 4th, 12th, and 16th weeks following BPAI, respectively. Images were acquired from a small animal PET/CT scanner (Concorde Microsystems, Knoxville, TN, USA).

Before PET/CT scanning, the rats stayed with water supply only for 12 h to enhance ^18^F-FDG uptake. ^18^F-FDG (0.5 m Ci) was injected through the tail vein, and then, the rat stayed in a quiet room for 30 min to ensure adequate take-up of tracer. Rats were induced with 5% halothane and maintained with 1.5%. The scan processes were conducted as previous study described (Shen et al., 2019a). During scanning, the rat was placed in prone position on the bed of PET/CT, which consisted of a 15-cm-diameter ring of 96-position sensitive ray scintillation detectors and provided a 10.8-cm transaxial and a 7.8-cm axial field of view (FOV) with an intrinsic resolution of 1.8 mm. The timing resolution was <1.5 ns. The CT images were obtained for coregistration and attenuation correction. The collected images were recombined in the OSEM3D mode with a 128 × 128 matrix. Parameters of CT image acquisition were set as follows: spherical tube voltage = 80 kV, current = 500 μA, and acquisition time = 492 s.

### Image Preprocessing

Data preprocessing was conducted using the Statistical Parametric Mapping 8 toolbox (SPM 8)^1^ based on Matlab 2014a (Mathworks, Inc., Natick, MA, USA). The raw PET images in DICOM format were converted into NIFTI format using ImageJ software (Image Processing and Analysis in Java, National Institutes of Health, Bethesda, MD, USA). The skull-stripped brain PET images of each rat were extracted by using a hand drawing mask. All the three directions (i.e., *x*, *y*, and *z*) of the voxels were upscaled by 10 to fit the algorithm implemented in SPM8. Based on a standard rat brain template (Schwarz et al., 2006), the orientation of individual PET images was adjusted by modifying pitch/roll/yaw parameters, and the origin was reset. As only one brain volume of standard uptake value (SUV) was obtained during one image acquisition period, the realignment procedure for correction of head motion among different volumes was inapplicable, thus not conducted. All individual brain PET images were then spatially normalized to the template and resampled to 2 × 2 × 2 mm^3^ resolution. The images were smoothed with a Gaussian kernel of a full-width at half-maximum (FWHM) twice of the voxel size (i.e., FWHM = 4 mm), to enhance the signal/noise ratio. The FDG uptake value of each voxel was normalized by the global mean uptake value to correct for variability across subjects.

### Metabolic Connectivity Construction

**Figure 2:**
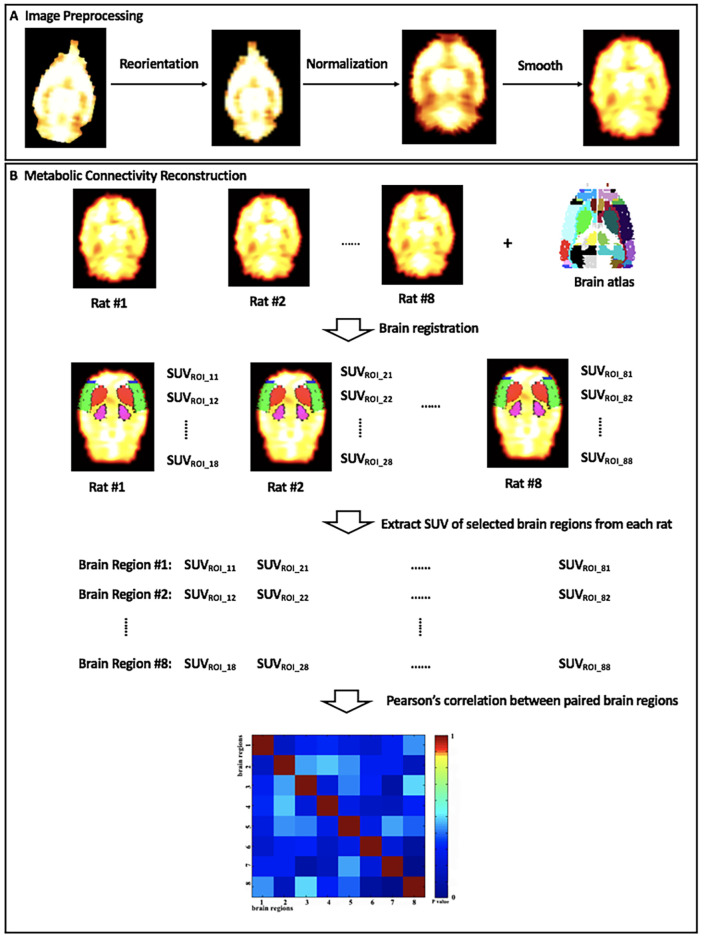
The diagram of **(A)** image preprocessing and **(B)** metabolic connectivity construction. SUV_ROI_*ij*_, the standard uptake value (SUV) of brain region #*j* in rat #*i*.

In the current study, a matrix of intersubject metabolic brain connectivity was constructed for each group of rats. The process is shown in Figure 2. In the matrix, a set of nodes represented selected brain regions, while their edges denoted functional relationship between nodes. Our previous report has revealed significantly altered glucose metabolism in bilateral motor cortex (MC), sensory cortex (SC), caudate putamen (Cpu), and dorsolateral thalamus (DLT) following BPAI (Shen et al., 2019a,b). Therefore, these above eight brain regions were defined as nodes, and they were drawn out by registering the stereotaxic rat brain atlas (Schwarz et al., 2006) with the normalized brain PET images of each rat. The mean SUV of each selected brain region from each rat was extracted and compiled into separate vectors. The cross-subject Pearson’s correlation coefficients between paired brain regions were calculated. Then, an adjacency 8 × 8 correlation matrix for each group was constructed with brain regions labeled on the *x*- and *y*-axes and *P*-values of correlation coefficients as the elements in rows and columns.

### Statistical Analysis

The SPSS 22.0 statistical software (SPSS Inc., New York, NY, USA) was used to analyze the behavioral data. The data were shown as mean ± standard deviation. Least significance difference (LSD) test was used to analyze the differences among the four groups at each time point. *P* < 0.05 were considered statistically significant.

After metabolic connectivity construction, a two-sample *t*-test was performed in the intragroup comparison of metabolic brain images (i.e., EA vs. sham EA, model vs. normal, sham EA vs. model, EA vs. model) with a significance level of *P* = 0.05 [false discovery rate (FDR) correction]. The analysis was performed using the Statistical Parametric Mapping 8 toolbox (SPM 8)^1^ based on Matlab 2014a (Mathworks, Inc., Natick, MA, USA).

## Results

### Animals

All rats were in good condition and active after BPAI modeling. They showed slight weakness within the first three postinjury days and then largely recovered to baseline level. No obviously abnormal foraging activity or instability was observed, and no infection was noted. Most rats in model, sham EA, and EA groups showed autotomy in the right (injured) forepaws, such as biting off the nails and digits. Horner’s sign (i.e., ptosis, concave eyeballs, and constriction of pupils) was presented on the right (injured) side in all rats.

### Behavioral Assessment

**Figure 3:**
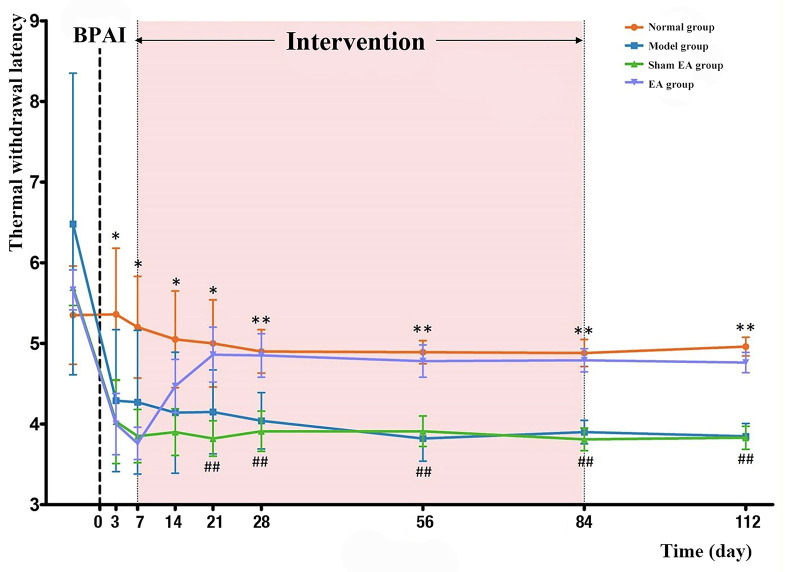
Comparison of TWL of the rats’ left (noninjured) forepaw in the normal, model, sham EA, and EA groups. BPAI was made on day 0. * and ** Indicate significant differences between the model and normal groups at the same timepoint (model–normal; *P < 0.05; **P < 0.001). ^##^Indicates significant differences between the EA and sham EA groups at the same timepoint (EA–sham EA; *P* < 0.001). BPAI, brachial plexus avulsion injury; EA, electroacupuncture.

Before BPAI, there was no significant difference in TWL assessments of the noninjured (left) forepaw among the four groups (*P* > 0.05). On the third day, and first and second weeks after BPAI, TWL of the model, sham EA, and EA groups significantly decreased compared with the normal group (*P* < 0.05). However, there was no significant difference between the sham EA and EA groups (*P* > 0.05). Since the third week after BPAI, the average TWL of the model group was still lower than that in the normal group (*P* < 0.001), and the TWL in the EA group started to be significantly higher than that in the sham EA group (*P* < 0.001). The changing curve is shown in Figure 3.

### Metabolic Brain Connectivity

**Figure 4:**
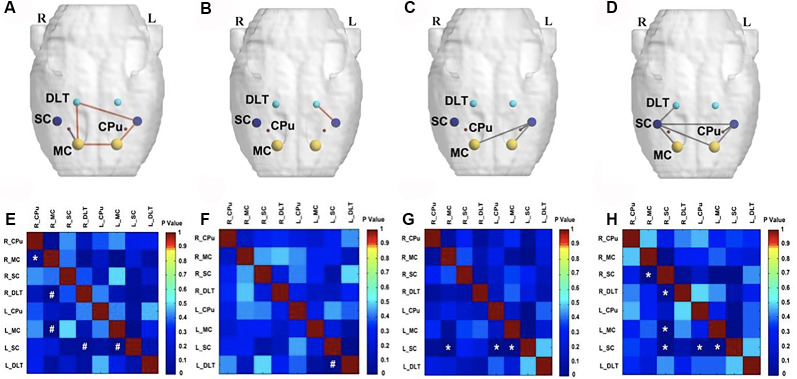
Comparison of metabolic connectivity among four groups at fourth week (i.e., 3 weeks of EA/sham EA intervention) after BPAI. **(A,E)** Significantly increased metabolic connectivity was observed among the right DLT, right motor cortex, left motor cortex, and left somatosensory cortex in the EA group, compared with the sham EA group. **(B,F)** Significantly increased metabolic connectivity was observed among the dorsolateral thalamus and left somatosensory cortex in the EA group, compared with the model. **(C,G)** Significantly increased metabolic connectivity among the left caudate putamen and left somatosensory cortex, as well as decreased metabolic connectivity between the right motor cortex and left somatosensory cortex, left motor cortex, and left somatosensory cortex was observed in the sham EA group, compared with the model group. **(D,H)** Changes in metabolic connectivity in the whole brain after BPAI (*P < 0.05; ^#^*P* < 0.001). Red line, increased metabolic connectivity; gray line, decreased metabolic connectivity. BPAI, brachial plexus avulsion injury; EA, electroacupuncture; SC, somatosensory cortex; DLT, dorsolateral thalamus; MC, motor cortex; CPu, caudate putamen.

On the fourth week following BPAI (i.e., 3 weeks of EA/sham EA intervention), significantly decreased metabolic connectivity was observed in bilateral motor, sensory, and pain-related brain regions (Figures 4D,H). Increased metabolic connectivity between regions related to motor and sensory regions in the right hemisphere, as well as decreased metabolic connectivity in the left hemisphere were observed in the sham EA group, compared with the model group (Figures 4C,G). However, significantly increased metabolic connectivity in bilateral hemispheres was observed in the EA group compared with the sham EA (Figures 4A,E) and model group (Figures 4B,F).

**Figure 5:**
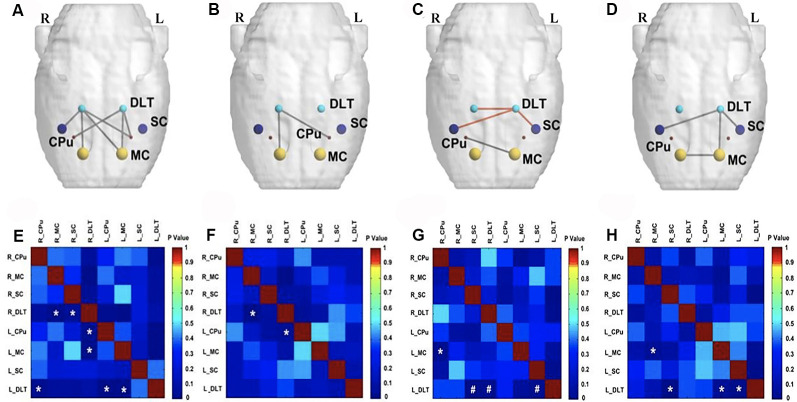
Comparison of metabolic connectivity among four groups at 12th week (i.e., 11 weeks of EA/sham EA intervention) after BPAI. **(A,E)** Significantly increased metabolic connectivity was observed among the bilateral DLT, bilateral motor cortex, bilateral caudate putamen, and right somatosensory cortices in the EA group compared with the sham EA group. **(B,F)** Significantly decreased metabolic connectivity was observed among the right DLT, right motor cortex, and left caudate putamen in the EA group compared with the model group. **(C,G)** Significantly increased metabolic connectivity between the left DLT and bilateral somatosensory cortices, as well as decreased metabolic connectivity between left motor cortex and right somatosensory cortex were observed in the sham EA group compared with the model group. **(D,H)** Changes of metabolic connectivity in the whole brain after BPAI (*P < 0.05; ^#^*P* < 0.001). Red line, increased metabolic connectivity; gray line, decreased metabolic connectivity. BPAI, brachial plexus avulsion injury; EA, electroacupuncture; SC. somatosensory cortex; DLT, dorsolateral thalamus; MC, motor cortex; CPu, caudate putamen.

At the end of the 12th week after BPAI (i.e., 11 weeks of EA/sham EA intervention), metabolic connectivity in bilateral hemispheres still decreased in the model group (Figures 5D,H). However, metabolic connectivity increased in the left hemisphere (contralateral to the injured limb) in the sham EA group (Figures 5C,G). Decreased metabolic connectivity in bilateral hemispheres was observed in the EA group compared with the sham EA group (Figures 5A,E) or with the model group (Figures 5B,F).

**Figure 6:**
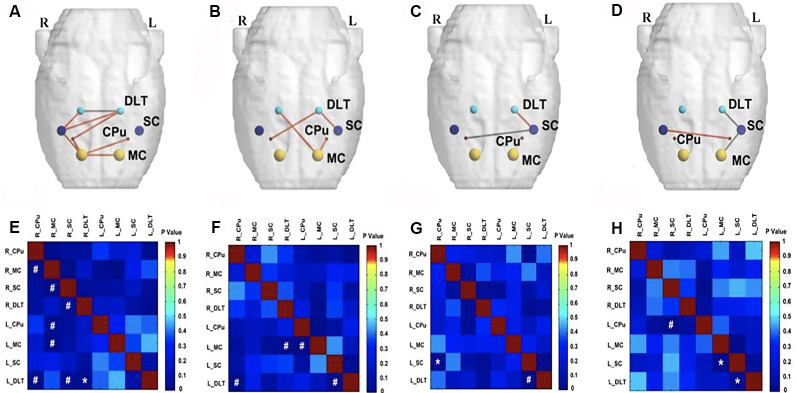
Comparison of metabolic connectivity among four groups at 16th week (i.e., 4 weeks after EA/sham EA intervention) after BPAI. **(A,E)** Significantly increased metabolic connectivity among the bilateral motor cortex, bilateral caudate putamen, bilateral DLT, and right somatosensory cortex, as well as significantly decreased metabolic connectivity between bilateral DLT were observed in the EA group compared with the sham EA group. **(B,F)** Significantly increased metabolic connectivity was observed among the bilateral DLT, left motor cortex, left somatosensory cortex, and right caudate putamen in the EA group compared with the model. **(C,G)** Significantly increased metabolic connectivity between the left DLT and left somatosensory cortex, as well as decreased metabolic connectivity between left somatosensory cortex and right caudate putamen were observed in the sham EA group compared with the model group. **(D,H)** Changes of metabolic connectivity in the whole brain after BPAI (*P < 0.05; ^#^*P* < 0.001). Red line, increased metabolic connectivity; gray line, decreased metabolic connectivity. BPAI, brachial plexus avulsion injury; EA, electroacupuncture; SC, somatosensory cortex; DLT, dorsolateral thalamus; MC, motor cortex; CPu, caudate putamen.

At the end of the 16th week after BPAI (i.e., 4 weeks after EA/sham EA intervention), increased metabolic connectivity was observed in bilateral hemispheres in the EA group compared with the sham EA group (Figures 6A,E) or the model group (Figures 6B,F). In the model (Figures 6D,H) and sham EA groups (Figures 6C,G), the changes were similar with those of previous timepoints (12 weeks).

## Discussion

Neuropathic pain is a persistent chronic pain that is associated with abnormal functioning caused by a lesion or disease affecting the somatosensory system (Treede et al., 2008). Many diseases could cause persistent chronic pain, such as diabetic peripheral neuropathy, nondiabetic neuropathy, postherpetic neuralgia, trigeminal neuralgia, and spinal cord injury (Bril et al., 2011; Finnerup et al., 2014; Mulla et al., 2015). BPAI is a specific type of brachial plexus injury that causes preganglionic disruption of nerve roots from the spinal cord (Murphey et al., 1947). High prevalence of neuropathic pain is also acknowledged in BPAI (Santana et al., 2016).

Although accumulating studies have focused on neuropathic pain (Zilliox, 2017), the treatment of neuropathic pain induced by BPAI is still challenging. For example, the American Academy of Neurology (ANN) has published practice guidelines including pharmacological and nonpharmacological treatment of painful diabetic neuropathy, postherpetic neuralgia, and trigeminal neuralgia (Gronseth et al., 2008; Bril et al., 2011). However, for neuropathic pain due to BPAI, reported guideline is still limited. Paszcuk et al. (2011) reported potential effect of cannabinoid and cannabinoid agonists in treating neuropathic pain following BPAI in mice (Paszcuk et al., 2011). However, the adverse effects associated with the use of “medical cannabis” and the safety of inhaling pyrolysis products in cannabis smoking process still need further investigation and improvement (Donvito et al., 2018). Previous study also demonstrated that motor cortex stimulation and deep brain stimulation may be effective for treating neuropathic pain following peripheral nerve injury (Levy et al., 2010). However, complications of the surgery, such as intracranial bleeding, infection, and permanent neurological deficits, need to be considered (Nguyen et al., 1999).

EA, originated from traditional acupuncture, is combined use of needles and electrical stimulation (Ju et al., 2017). Cope (2016) reported that repeated EA contributes to significant analgesic effects in treating brachial plexus neuralgia induced by administration of cobra venom (Cope, 2016). Zhang et al. (2014) reported that EA intervention can relieve neuropathic pain in BPAI patients.

Therefore, we established the neuropathic pain model by BPAI. Thermalgesia threshold of the noninjured forepaw was significantly decreased after BAPI, which is consistent with our previous studies. We found that thermalgesia threshold of the noninjured forepaw was elevated after application of 2/15 Hz EA stimulation in the “Jiaji” acupoints (EX-C5–C7). These demonstrated that EA could potentially release neuropathic pain following BPAI, and the treatment may persist after the cease of intervention.

The mechanism of neuropathic pain in BPAI is complicated. In BPAI, both peripheral nerve fibers and spinal cord were injured (Teixeira et al., 2015). Thus, several neural pathways are involved in the occurrence and development of neuropathic pain following BPAI. Peripheral nerve injury leads to sensitization of primary afferents inputs *via* the changes in the expression of neurotransmitters, neuromodulators, growth factors, and neuroactive molecules located in the dorsal root ganglion (Jaggi and Singh, 2011). Aside from that, BPAI also leads to hyperactivity of neuron in the posterior horn of the spinal cord (Denny-Brown et al., 1973; Powers et al., 1984), resulting in sensitization in the spinal cord (Jaggi and Singh, 2011). With the development of brain imaging and electrophysiological technology, a number of studies have reported that the brain has a network of cortical and subcortical areas that constitutes the “pain matrix” (Moisset and Bouhassira, 2007; Iannetti and Mouraux, 2010) that are associated with the sensory-discriminative (Hsieh et al., 1996; Apkariana et al., 2005; Moisset and Bouhassira, 2007), affective-emotional (Hsieh et al., 1996; Moisset and Bouhassira, 2007), and cognitive aspects (Apkariana et al., 2005; Moisset and Bouhassira, 2007) of pain. The “pain matrix” may provide insight of cerebral mechanism of neuropathic pain following BPAI.

There also have been some researches in the mechanism of acupuncture/EA in treating neuropathic pain. Li et al. (2019) found that 6 weeks of EA intervention could activate μ-opioid receptors, inhibit spinal dorsal horn neuron, and thus release pain. Low frequency of EA inhibits neuropathic pain more effectively than high frequency. It is reported that 2 Hz of EA induced a robust and longer-lasting decreased mechanical allodynia threshold than 100 Hz in caudal trunk nerve injury-induced neuropathic pain (Kim et al., 2004). In an L5/L6 nerve ligation-induced neuropathic pain model, 2 Hz also decreased mechanical and thermal hypersensitivity more powerfully than 100 Hz (Sun et al., 2002). Aside from that, spinal serotonin and norepinephrine participate in EA inhibition of neuropathic pain. EA can activate 5-HT1ARs to inhibit *N*-methyl-D-aspartate receptor (NMDAR) activities (Zhang et al., 2014). Park et al. (2010) also found that EA could reduce the release of excitatory amino acids and promote the release of inhibitory amino acid neurotransmitters in spinal cord (Park et al., 2010). Jiang et al. (2016) found that EA could inhibit secretion of prostaglandin E2 in the spinal cord and reduce pain by inserting bilateral L5 “Jiaji” (EX-B2), “Dachangshu” (BL25), “Weizhong” (BL40), and “Kunlun” (BL60) acupoints in rats. Other mechanisms include inhibiting of glial cells and inducing of numerous bioactive chemicals in the spinal cord (Zhang et al., 2014). Zhang et al. reported that EA stimulation could attenuate neuropathic pain after brachial plexus injury through upregulating β-endorphin expression (Zhang et al., 2014). Our previous studies revealed that application of EA induced activation and subsequent deactivation of somatosensory area and pain-related regulating cortical areas, including insula, thalamus, and cingulate cortex in a sciatic nerve transection model (Wu et al., 2018b). We also found that EA could induce both regional and extensive neuroplasticity in bilateral hemispheres (Wu et al., 2018a).

^18^FDG-PET has been widely used to evaluate regional brain glucose metabolic connectivity in many diseases to provide further understanding of neural functional connectivity (Morbelli et al., 2012; Caminiti et al., 2016; Pagani et al., 2016; Herholz et al., 2018). Horwitz et al. (1987) suggested that metabolic connectivity was the across-subject correlations of glucose metabolism between different brain regions (Horwitz et al., 1987). The altered metabolic connectivity revealed the changes in brain functional network, which was associated with pathophysiology of disorders. Our previous study also showed that neuropathic pain following BPAI induced metabolic connectivity changes significantly among sensorimotor-related areas and pain-related area in bilateral hemispheres (Huo et al., 2020). The specific neuroplasticity pattern might provide further insights to the mechanism of neuropathic pain following BPAI.

In the present research, we used ^18^FDG-PET to assess the changes of metabolic connectivity in neuropathic pain following BPAI. We found that decreased metabolic connectivity between bilateral sensorimotor cortices from 4th to 14th weeks. The decreased activation of sensorimotor cortex (SMC) could be attributable to the lack of neural input signals from periphery (Yoshikawa et al., 2012). We noted increased metabolic connectivity between SMC and pain-related areas in bilateral hemispheres at 4th week and decreased metabolic connectivity at 12th week following EA intervention. However, at 16th week, increased metabolic connectivity was found between SMC and pain-related areas in bilateral hemisphere. The specific activation pattern may be associated with the central mechanism of EA intervention in treating neuropathic pain after BPAI.

Caudate and putamen, which constitute the dorsal striatum, are the gateway to the basal ganglia (Kreitzer and Malenka, 2008). The Cpu receives inputs from all cortical areas and projects to frontal lobe areas (prefrontal, premotor, and supplementary motor areas) throughout the thalamus, which are concerned with motor planning (Herrero et al., 2002). Lanz et al. (2011) reported that increased activity of Cpu was associated with sensation of thermal pain. In the present experiment, the decreased metabolic connectivity between SC and Cpu in the left hemisphere (contralateral to the injured forelimb) may be the part explanation of the neuroplasticity evoked by chronic neuropathic pain and the dysfunction of motor and sensory function.

The thalamus is a key relay station for the transmission of nociceptive information to the cerebral cortex. It receives somatosensory inputs from the skin, deep structures, and visceral organs and then projects to the cortex. The dorsal thalamus contains the principal somatosensory thalamic nuclei (Yen and Lu, 2013). Previous studies have revealed the importance of thalamus in neuromodulation of chronic pain. Iadarola et al. (1995) reported reduced regional blood flow in the thalamus contralateral to the injured limb in patients with spontaneous pain. Application of deep brain stimulation in thalamus was demonstrated to be efficient for release of chronic neuropathic pain after traumatic amputation and BPAI (Pereira et al., 2013). In our study, decreased metabolic connectivity between ipsilateral DLT and SC was related with BPAI-induced neuropathic pain.

However, the present study still has some limitations. The natural difference between human and rodents should not be neglected. The acupoints are empirical proof in human that need further evidence. Further researches in human and brain microstructures are still needed before full confirmation is made.

## Data Availability Statement

The raw data supporting the conclusions of this article will be made available by the authors, without undue reservation.

## Ethics Statement

All procedures were in agreement with the Guide for the Care and Use of Laboratory Animals described by the US National Institutes of Health and were reviewed and approved by the Animal Ethical Committee of Shanghai University of Traditional Chinese Medicine.

## Author Contributions

A-LH and B-BH designed the study and monitored the progress, data collection, and analysis. M-XZ and X-YH analyzed and interpreted the data and made critical revision of the article. JS and X-YH designed the study and collected the data. J-GX supervised the progress of the study.

## Conflict of Interest

The authors declare that the research was conducted in the absence of any commercial or financial relationships that could be construed as a potential conflict of interest.

## Footnotes

^1^http://www.fil.ion.ucl.ac.uk/spm/
