# Supplementary material for: Electroacupuncture-Related Metabolic Brain Connectivity in Neuropathic Pain due to Brachial Plexus Avulsion Injury in Rats
Source: Front Neural Circuits. 2020 Jun 17;14:35. doi: 10.3389/fncir.2020.00035 (PMC7313422; doi:10.3389/fncir.2020.00035)
Supplement: Supplementary file 1 [file Data_Sheet_1.PDF]

Supplementary Materials

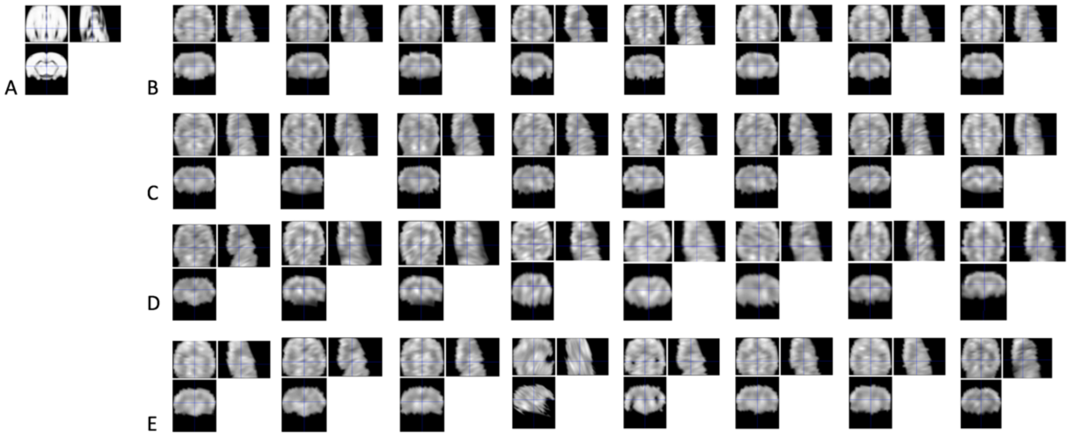

Figure S1. The results of normalization on the 4th week following brachial plexus avulsion injury (BPAI) (i.e., 3 weeks of EA/sham EA intervention).

(A) The rat brain template used for normalization. (B~E) The post-normalization brain images of rats in EA (B), sham EA (C), normal (D), and BPAI model (E) groups.

EA: electroacupuncture

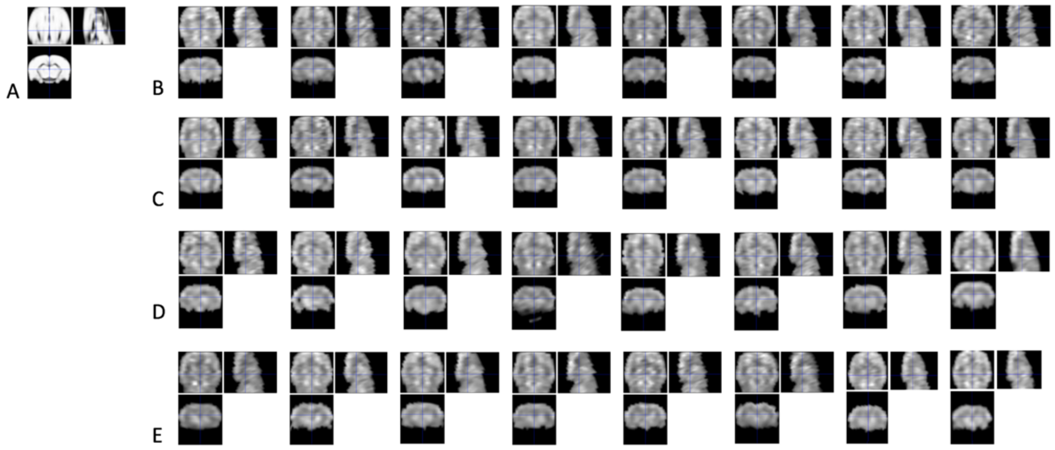

Figure S2. The results of normalization on the 12th week following brachial plexus avulsion injury (BPAI) (i.e., 11 weeks of EA/sham EA intervention).

(A) The rat brain template used for normalization. (B~E) The post-normalization brain images of rats in EA (B), sham EA (C), normal (D), and BPAI model (E) groups.

EA: electroacupuncture

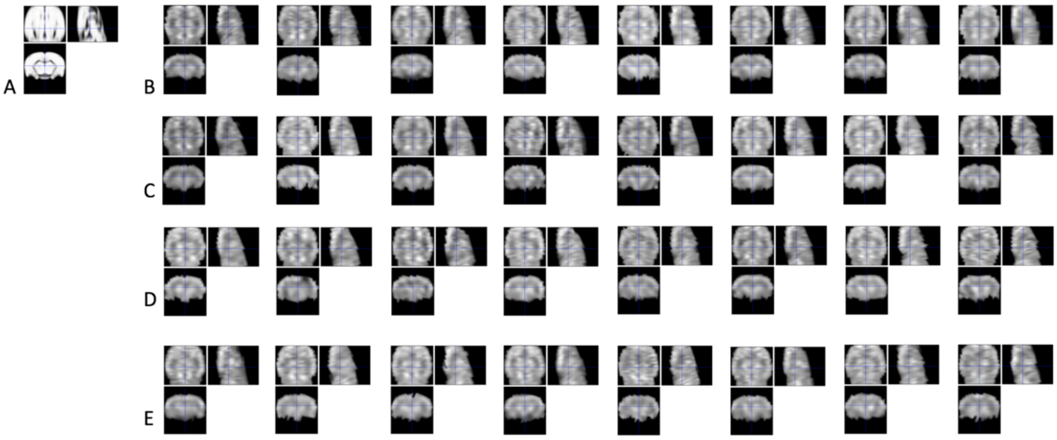

Figure S3. The results of normalization on the 16th week following brachial plexus avulsion injury (BPAI) (i.e., 4 weeks after EA/sham EA intervention).

(A) The rat brain template used for normalization. (B~E) The post-normalization brain images of rats in EA (B), sham EA (C), normal (D), and BPAI model (E) groups.

EA: electroacupuncture
